# Molecular Insights into β-Cyclodextrin-Based Supramolecular Deep Eutectic Solvent Formation and Its Interactions with Curcumin

**DOI:** 10.3390/molecules31213844

**Published:** 2026-10-25

**Authors:** Damen Nurgaliyeva, Mirat Karibayev, Galiya Baisalova, Anuar Aldongarov, Aitkazy Kaisha, Zhaksylyk Suiindik, Aizhan Zeinuldina, Yerbolat Kalpakov, Sarzhan Sharipova, Kulzada Bazhykova, Sergei Piskunov

**Affiliations:** 1 Department of Natural Sciences, Astana International University, Astana 010000, Kazakhstan; damennurgalieva@gmail.com; 2 National Laboratory Astana, Nazarbayev University, Astana 010000, Kazakhstan; mirat.karibayev@nu.edu.kz (M.K.); aitkazy.kaisha@nu.edu.kz (A.K.); 3 Department of Chemistry, L.N. Gumilyov Eurasian National University, Astana 010000, Kazakhstan; bajsalovagalia1@gmail.com (G.B.); enu-2010@yandex.kz (A.A.); 4 Department of Chemistry, Nazarbayev University, Astana 010000, Kazakhstan; zhaksylyk.suiindik@nu.edu.kz; 5 Department of General and Biological Chemistry, National Medical University Named After Toregeldy Sharmanov, Astana 010000, Kazakhstan; 6 School of Intelligent Systems, Astana IT University, EXPO C1, Mangilik El 55/11, Astana 010000, Kazakhstan; y.kalpakov@astanait.edu.kz; 7 Department of Pharmaceutical and Toxicological Chemistry, Asfendiyarov Kazakh National Medical University, Almaty 050012, Kazakhstan; 8 Department of Chemistry and Technology of Organic Substances, Natural Compounds and Polymers, Al-Farabi Kazakh National University, Almaty 050000, Kazakhstan; 9 Institute of Solid State Physics, University of Latvia, LV-1063 Riga, Latvia

**Keywords:** curcumin, supramolecular deep eutectic solvents, β-cyclodextrin, choline chloride, glycerol, molecular dynamics

## Abstract

This study investigates the molecular organization and interaction behavior of curcumin in a choline chloride (ChCl):glycerol (Gly):β-cyclodextrin (βCD) supramolecular deep eutectic solvent (supraDES) using classical all-atom molecular dynamics (MD) simulations. The formation of the βCD-containing supraDES and its interactions with curcumin were analyzed mainly using molecular snapshots, radial distribution functions (RDFs), hydrogen bond analysis, interaction energies, density profiles, and root-mean-square deviation (RMSD). Regarding supraDES formation, molecular structure, RDF, hydrogen bond, and interaction energy analyses revealed that the incorporation of βCD modifies the local coordination environment of the DES components. Upon curcumin addition, a correlation between curcumin and βCD was observed, indicating a preferential association between curcumin and the supramolecular host, as revealed by the analysis of classical all-atom MD simulation outputs. Future studies should combine MD simulations with experimental investigations and enhanced free-energy calculations to further elucidate the host–guest binding mechanisms and assess the potential of βCD-based supraDESs for curcumin solubilization and delivery.

## 1. Introduction

Curcumin, the principal bioactive polyphenol of *Curcuma longa* L. (turmeric), exhibits antioxidant, anti-inflammatory, anticancer, antimicrobial and neuroprotective activities, which make it an attractive candidate for pharmaceutical, food and biomedical applications [1,2,3]. Its use, however, is severely limited by very low water solubility (approximately 1 μg/mL) and pronounced chemical instability toward light, heat and pH, which together lead to poor oral bioavailability [3,4,5].

Deep eutectic solvents (DES), formed from natural hydrogen-bond donors and acceptors such as ammonium salts, organic acids, sugars and polyols, have emerged as biodegradable, low-volatility and tunable green solvents capable of dissolving both hydrophilic and hydrophobic bioactive compounds, including curcuminoids [6,7,8,9,10,11]. SupraDES go a step further by incorporating host molecules, most notably cyclodextrins, into the eutectic matrix [12,13,14]. The βCD, with a hydrophilic exterior and a lipophilic cavity, can include hydrophobic curcuminoids and thereby combine efficient extraction with a protective microenvironment [13,14,15,16,17,18,19,20].

Host-guest complexation by cyclodextrins is, however, strongly governed by the structure of the host and by the ionization state of the guest. Garibyan et al. showed that βCD is a more effective solubilizer of baricitinib than α- and γ-CD and binds its neutral form two to three times more strongly than its cationic form, resulting in a three-fold solubility increase at pH 6.8 but only a 1.5-fold increase at pH 1.6 [21]. Comparable trends from previous experimental studies are summarized in Table 1. Cavity size determines binding strength and stoichiometry. Namely, β- and γ-CD bind amino acids better than α-CD [22], α-CD alone forms 2:1 complexes with n-alkanes [23], and the binding of a coumarin dye increases from β- to γ-CD [24]. Methylated and acetylated derivatives often enhance complexation [25,26], although native βCD outperformed HP-β-CD for piroxicam [27]; sulfobutylether with β-CD gave the largest solubility gains for dofetilide and tyrosine kinase inhibitors [28,29], while dendrimers outperformed cyclodextrins for niclosamide [30]. Guest ionization generally increases hydration and weakens inclusion, as described by the pH-dependent stability-constant model of Samuelsen et al. [31] and observed for ketoprofen, aspirin and aminobenzoic acids [32,33,34]. Nevertheless, complexation often persists for charged guests [26,28,35], and ionizable groups on the host can be used to switch binding [36].

Almost all of these data were obtained in dilute aqueous buffers, and how cavity fit and charge operate in a crowded, hydrogen-bonded eutectic medium remains unexplored. Aimone et al. recently reported a ChCl: Gly: βCD based supraDES (1:2:0.02 molar ratio, 10% *w*/*w* water) that improved curcuminoid extraction from *C. longa* and reduced 30-day degradation from 62.8% (βCD-free ChCl:Gly DES) to 47.5% [15]. The molecular organization of this system, including whether curcumin is included in βCD, how the DES components compete for the cavity and rims, and how curcumin is solvated, has not been characterized. Because curcumin degradation involves bond-breaking reactions occurring over days to weeks [2,3], it cannot be described by classical force fields on nanosecond timescales. Classical all-atom MD simulations are, however, well suited to resolving the molecular structure, hydrogen bonding, RDF, and interaction energetics of such systems [16,17,18,19,20].

In this study, we use classical all-atom MD simulations to examine the formation of the ChCl:Gly:βCD based supraDES and the interactions of curcumin with βCD and the individual DES components. Rather than modeling degradation, the simulations identify structural and intermolecular interaction features that can guide the rational design of supraDES media for curcumin and other hydrophobic bioactive compounds.

## 2. Results and Discussion

### 2.1. Formation of supraDES

#### 2.1.1. Molecular Structures

**Figure 1:**
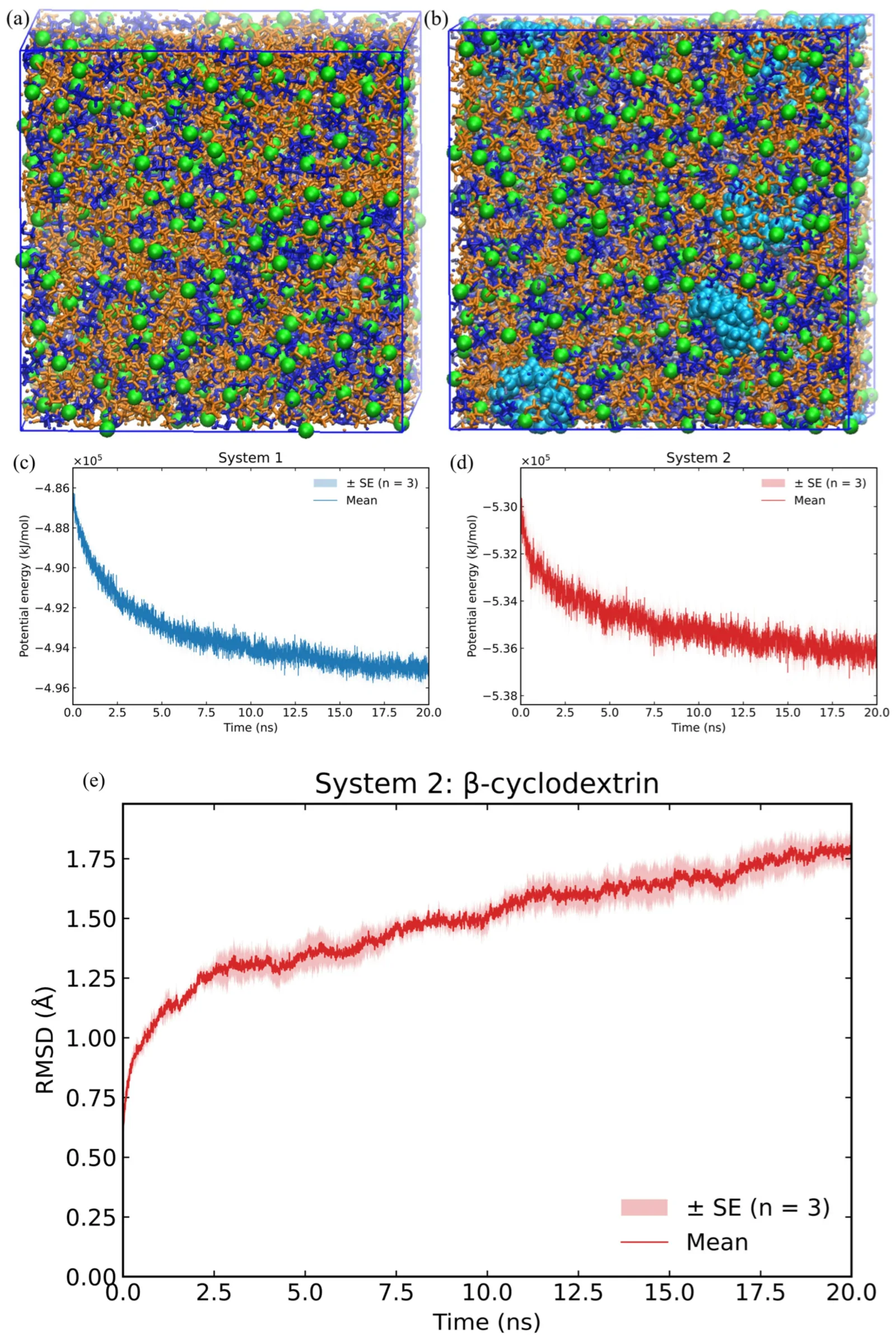
Molecular snapshots of (**a**) ChCl:Gly based DES and (**b**) ChCl:Gly: βCD:10% water based supraDES. Schematic representation and color key: Licorice representation and blue (color ID: 24) molecules (ChCl); Licorice representation and orange (color ID: 3) molecules (Gly); Van der Waals surface representation and green (color ID: 7) (Chloride ion); Licorice representation and ice blue (color ID: 15, transparent) molecules (Water), and Van der Waals surface representation and cyan (color ID: 22) (βCD). (**c**) Potential energy of system 1, (**d**) Potential energy of system 2, (**e**) RMSD of βCD over 20 ns of classical all-atom MD production run.

Figure 1a,b shows representative molecular snapshots of the ChCl:Gly DES (system 1) and of the ChCl:Gly:βCD supraDES containing 10% (*w*/*w*) water (system 2) at the end of the 20 ns production run. In system 1, choline cations, glycerol molecules and chloride anions are homogeneously intermixed, and no segregated domains of any component are visible, as expected for a fully mixed, extensively hydrogen-bonded eutectic liquid. In system 2, the βCD molecules are distributed throughout the simulation box and are surrounded by choline, glycerol and chloride, while the water molecules are dispersed within the DES network rather than forming a separate aqueous phase. No macroscopic phase separation was observed in either system.

Molecular snapshots provide only a qualitative, single-frame view of the systems. The convergence of the simulations and the structure of the liquids were therefore characterized quantitatively using potential energies, βCD RMSD, RDFs, hydrogen-bond numbers and short-range interaction energies, each averaged over three independent replicas (Section 2.1.2, Section 2.1.3, Section 2.1.4, Section 2.1.5 and Section 2.1.6).

#### 2.1.2. Potential Energies

Figure 1c,d shows the time evolution of the potential energy of systems 1 and 2 averaged over three independent replicas (shaded band: ±SE). In both systems, the potential energy decreases during approximately the first 10 ns of the production run, reflecting the slow structural relaxation of the viscous, hydrogen-bonded DES network after the short NVT/NPT equilibration, and then levels off. Over the last 5 ns (15–20 ns), the average potential energies were −494,954 ± 205 kJ/mol (system 1) and −536,054 ± 376 kJ/mol (system 2). Within this window, the residual drift was −54 and −74 kJ/mol per ns, respectively, i.e., less than 0.015% of the total potential energy per ns, and the averages of the first and second halves of the window differed by less than 0.03%.

The standard errors between replicas were below 0.1% of the total potential energy, showing that the three independent trajectories of each system reach the same energetic state. The first 15 ns of each production run were therefore treated as additional equilibration, and all structural and energetic analyses reported below were performed over the last 5 ns. Because systems 1 and 2 differ in composition and number of atoms (Section 3), their absolute potential energies are not directly comparable; the more negative value of system 2 mainly reflects its additional βCD and water molecules. We also note that 20 ns is short compared with the structural relaxation times of DES at room temperature. The present analysis therefore focuses on local structure and short-range interactions, which converge faster than collective and transport properties; the latter were not evaluated.

#### 2.1.3. RMSD

The structural stability of βCD in the supraDES was assessed from its RMSD relative to the starting structure (Figure 1e). The RMSD increases rapidly to approximately 1.25 Å within the first 2.5 ns and then rises slowly, reaching 1.72 ± 0.07 Å on average over the last 5 ns, with a residual slope of approximately 0.03 Å/ns. These deviations are small compared with the dimensions of the βCD macrocycle (outer diameter of approximately 15 Å), indicating that βCD retains its overall ring conformation in the supraDES.

The slow increase in RMSD is most likely due to local conformational adjustments of the glucopyranose units and their hydroxyl groups to the surrounding DES environment, rather than to a loss of the ring structure. The small standard errors (≤0.07 Å) show that the three replicas follow the same behaviour. The RMSD of βCD in the curcumin-containing systems is discussed in Section 2.2.3.

#### 2.1.4. RDFs

**Figure 2:**
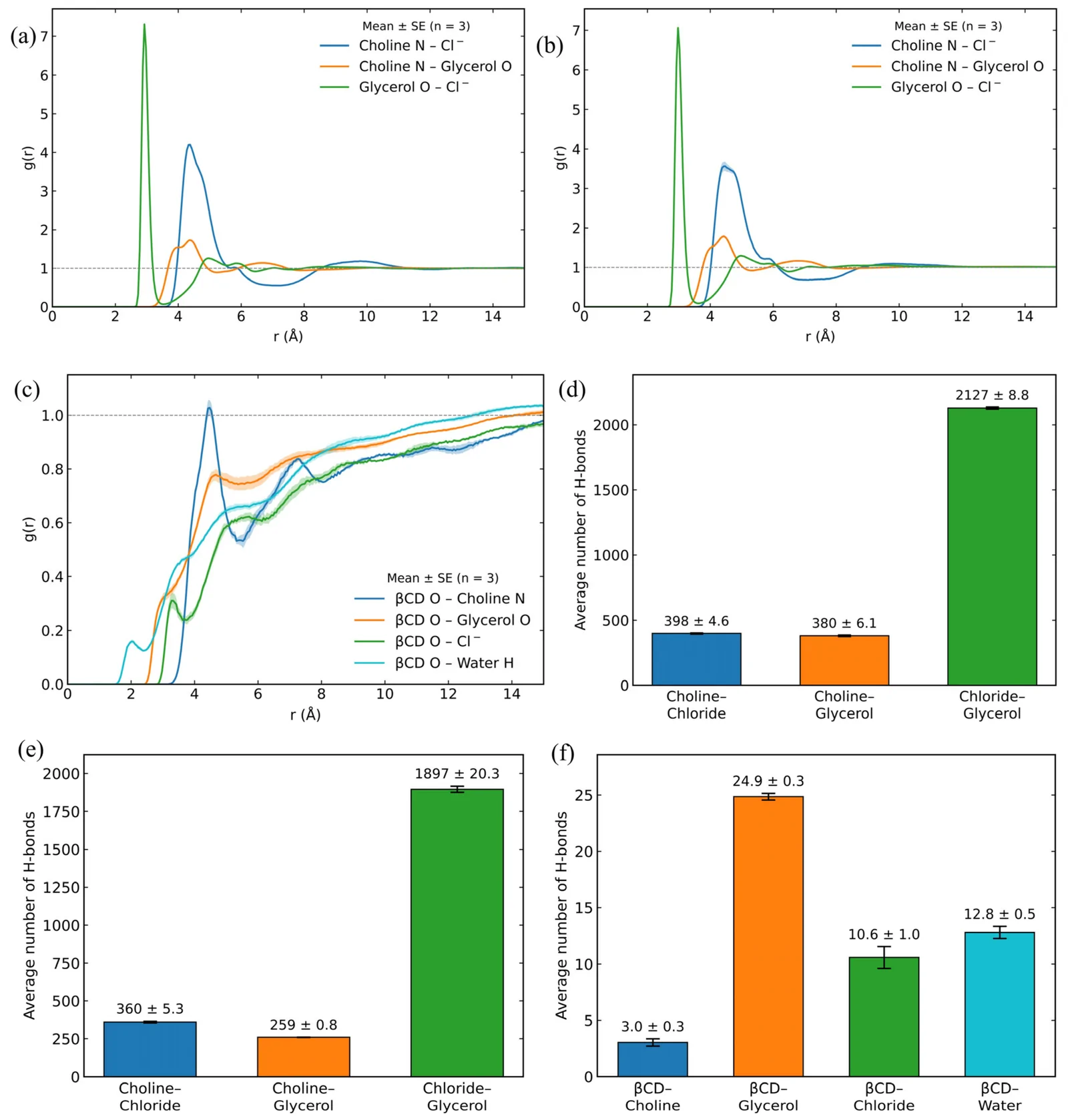
(**a**) RDFs between choline and chloride, choline and glycerol, and chloride and glycerol in the DES (system 1). (**b**) RDFs between choline and chloride, choline and glycerol, and chloride and glycerol in the supraDES (system 2). (**c**) RDFs between βCD and choline, glycerol, chloride ions, and water molecules in the supraDES (system 2). (**d**) Number of hydrogen bonds between choline and chloride, choline and glycerol, and chloride and glycerol in the DES (system 1). (**e**) Number of hydrogen bonds between choline and chloride, choline and glycerol, and chloride and glycerol in the supraDES (system 2). (**f**) Number of hydrogen bonds between βCD and choline, glycerol, chloride ions, and water molecules in the supraDES (system 2). Analyses were performed over last 5 ns of classical all-atom MD production run.

Figure 2a,b shows the RDFs between the choline nitrogen (ChN), the glycerol hydroxyl oxygens (GlyO) and the chloride anions in systems 1 and 2. All RDFs were computed up to 15 Å and converge to unity at long range, confirming their normalization. To complement the peak heights, coordination numbers (CN) were obtained by integrating the RDFs up to their first minimum.

In the DES (system 1), the GlyO–Cl^−^ RDF shows the sharpest and most intense first peak, at 2.93 Å (g(r) = 7.31 ± 0.03), followed by a first minimum at 3.53 Å (CN = 0.49 chloride per glycerol oxygen). This distance is characteristic of O–H···Cl^−^ hydrogen bonds, which therefore constitute the dominant short-range structural motif of the DES. The ChN–Cl^−^ RDF has a broader first peak at 4.38 Å (g(r) = 4.20 ± 0.01) and a first minimum at 7.18 Å, corresponding to approximately four chloride anions (CN = 4.00) in the first coordination shell of each choline cation. Because the quaternary nitrogen is shielded by its methyl groups, this peak reflects cation–anion packing rather than hydrogen bonding at the nitrogen. The GlyO–ChN correlation is comparatively weak, with a first peak at 4.38 Å (g(r) = 1.73 ± 0.01), a shoulder near 4.0 Å, and CN = 1.23 up to 5.23 Å.

In the supraDES (system 2), all peaks appear at essentially the same positions (2.98, 4.48 and 4.43 Å for GlyO–Cl^−^, ChN–Cl^−^ and GlyO–ChN, respectively), showing that the main structural motifs of the DES are preserved. However, the first-shell coordination is reduced: the GlyO–Cl^−^ peak decreases to 7.06 ± 0.12 (CN = 0.44, −10%), the ChN–Cl^−^ peak to 3.56 ± 0.12 (CN = 3.52, −12%), and the GlyO–ChN CN to 1.11 (−10%), while the GlyO–ChN peak height is practically unchanged (1.79 ± 0.03). Because βCD and water were introduced together, these changes reflect their combined effect and cannot be assigned to βCD alone. They are consistent with water molecules and βCD partly replacing DES–DES contacts in the first coordination shells.

The RDFs between βCD and the supraDES components (Figure 2c) were computed using the oxygen atoms of βCD (hydroxyl and glycosidic oxygens) as reference and the choline nitrogen, glycerol oxygens, chloride and water hydrogens as targets. Because βCD is a large macrocycle that excludes a considerable volume around many of its oxygen atoms, these RDFs remain below or close to unity at short range and approach unity only beyond approximately 12 Å. Their peak heights are therefore not interpreted as strong associations, and the discussion focuses on peak positions. The shortest-range feature is the βCD O–water H peak at approximately 2.0 Å (g(r) ≈ 0.16), corresponding to water hydrogen bonded to βCD oxygens. The βCD O–glycerol O RDF shows a shoulder at approximately 2.8 Å (g(r) ≈ 0.3), which is within the O···O hydrogen-bond range, and the βCD O–Cl^−^ RDF shows a small peak at approximately 3.3 Å (g(r) ≈ 0.31), consistent with O–H···Cl^−^ contacts. The βCD O–choline N RDF displays its main peak at 4.45 Å (g(r) = 1.03 ± 0.03), which reflects packing of the choline head group against βCD rather than hydrogen bonding. Overall, βCD is solvated by all supraDES components, with only weak structural preferences in the RDFs; these interactions are quantified directly by hydrogen-bond counts and interaction energies below.

#### 2.1.5. Number of Hydrogen Bonds

Hydrogen bonds were identified using geometric criteria (donor–acceptor distance ≤ 3.5 Å and donor–hydrogen···acceptor angle ≥ 150°) and averaged over the last 5 ns of each replica (Figure 2d–f). In the DES (system 1), the average numbers of hydrogen bonds were 2127.5 ± 8.8 for glycerol–chloride, 397.9 ± 4.6 for choline–chloride and 379.9 ± 6.1 for choline–glycerol. Normalized per molecule, this corresponds to 2.84 hydrogen bonds per chloride (1.42 per glycerol) for glycerol–chloride, 0.53 per choline for choline–chloride and 0.25 per glycerol for choline–glycerol. The glycerol–chloride count agrees well with the GlyO–Cl^−^ coordination number from the RDF (0.49 × 4500 glycerol oxygens/750 chlorides ≈ 2.9 per chloride), confirming that the first GlyO–Cl^−^ RDF peak corresponds to hydrogen-bonded pairs.

In the supraDES (system 2), the corresponding numbers decreased to 1896.8 ± 20.3 (glycerol–chloride, −10.8%), 360.3 ± 5.3 (choline–chloride, −9.4%) and 259.3 ± 0.8 (choline–glycerol, −31.7%). The choline–glycerol hydrogen bonds are the most affected, while the glycerol–chloride network, the backbone of the DES, is largely retained (2.53 hydrogen bonds per chloride). These results confirm the trends in the RDFs: the hydrogen-bond network of ChCl:Gly persists in the supraDES but is partially disrupted by the combined presence of water and βCD.

For βCD in system 2 (Figure 2f), glycerol is by far the main hydrogen-bonding partner (24.9 ± 0.3 hydrogen bonds), followed by water (12.8 ± 0.5), chloride (10.6 ± 1.0) and choline (3.0 ± 0.3). Per βCD molecule, this corresponds to 1.66, 0.85, 0.71 and 0.20 hydrogen bonds, respectively. Thus, although the supraDES contains 10% (*w*/*w*) water, the βCD hydroxyl groups interact preferentially with glycerol, which is the most abundant hydroxyl-rich component. Choline forms few hydrogen bonds with βCD, consistent with the choline–βCD RDF peak at 4.45 Å, which indicates that choline contacts βCD mainly through van der Waals packing.

#### 2.1.6. Interaction Energies

The average short-range electrostatic (Coulomb) and van der Waals (Lennard-Jones) interaction energies between the components were obtained from the energy-group decomposition of the trajectories over the last 5 ns and are reported as mean ± SE of three replicas (Table 2 and Table 3). These values are total energies between all molecules of the two groups within the 1.0 nm cut-off; they are not normalized per molecule and do not include the reciprocal-space part of the PME electrostatics. The values for different pairs therefore reflect both the strength of the interactions and the number of molecules involved. They are used here to compare the same pair between systems, and they should not be interpreted as binding free energies.

In the DES (system 1), the chloride–glycerol pair has the most negative electrostatic energy (−153,273 ± 359 kJ/mol), followed by choline–chloride (−85,448 ± 290 kJ/mol) and choline–glycerol (−33,898 ± 292 kJ/mol), in line with the hydrogen-bond numbers. Choline–glycerol is the only DES pair with a substantial attractive van der Waals contribution (−33,746 ± 126 kJ/mol). The positive van der Waals energies of chloride–glycerol (20,817 ± 54 kJ/mol) and choline–chloride (1699 ± 90 kJ/mol) arise from the repulsive part of the Lennard-Jones potential at the short contact distances of hydrogen-bonded pairs. In the GROMOS force field, hydroxyl hydrogens carry no Lennard-Jones parameters, so the oxygen and chloride atoms approach each other closely. This repulsion is outweighed by the much larger electrostatic attraction.

In the supraDES (system 2), all DES–DES interaction energies become less negative: the electrostatic energies decrease in magnitude by 31% (choline–glycerol), 18% (choline–chloride) and 15% (chloride–glycerol), and the choline–glycerol van der Waals energy by 16% (Table 2). At the same time, the same energy-group decomposition shows substantial interactions of the DES components with water (e.g., electrostatic energies of approximately −58,750 kJ/mol for glycerol–water and −50,020 kJ/mol for chloride–water; not tabulated). Together with the RDF and hydrogen-bond results, this indicates that water and βCD partly replace DES–DES contacts. As discussed above, this is the combined effect of βCD and water incorporation.

The interactions of βCD with the supraDES components are summarized in Table 3. Glycerol shows the most favourable total interaction with βCD (−1010.1 ± 21.9 kJ/mol electrostatic and −3273.3 ± 139.5 kJ/mol van der Waals), followed by choline (−410.2 ± 10.2 and −2073.9 ± 43.4 kJ/mol), chloride (−1166.4 ± 62.2 and −235.1 ± 0.5 kJ/mol) and water (−532.9 ± 27.0 and −374.6 ± 11.0 kJ/mol). Per βCD molecule, the total short-range interaction energies are approximately −286 (glycerol), −166 (choline), −93 (chloride) and −61 kJ/mol (water). The βCD–choline interaction is dominated by van der Waals contributions, whereas the βCD–chloride interaction is predominantly electrostatic. The ranking agrees with the hydrogen-bond analysis and shows that βCD in the supraDES is solvated mainly by glycerol.

### 2.2. SupraDES Interaction with Curcumin

#### 2.2.1. Molecular Structures

**Figure 3:**
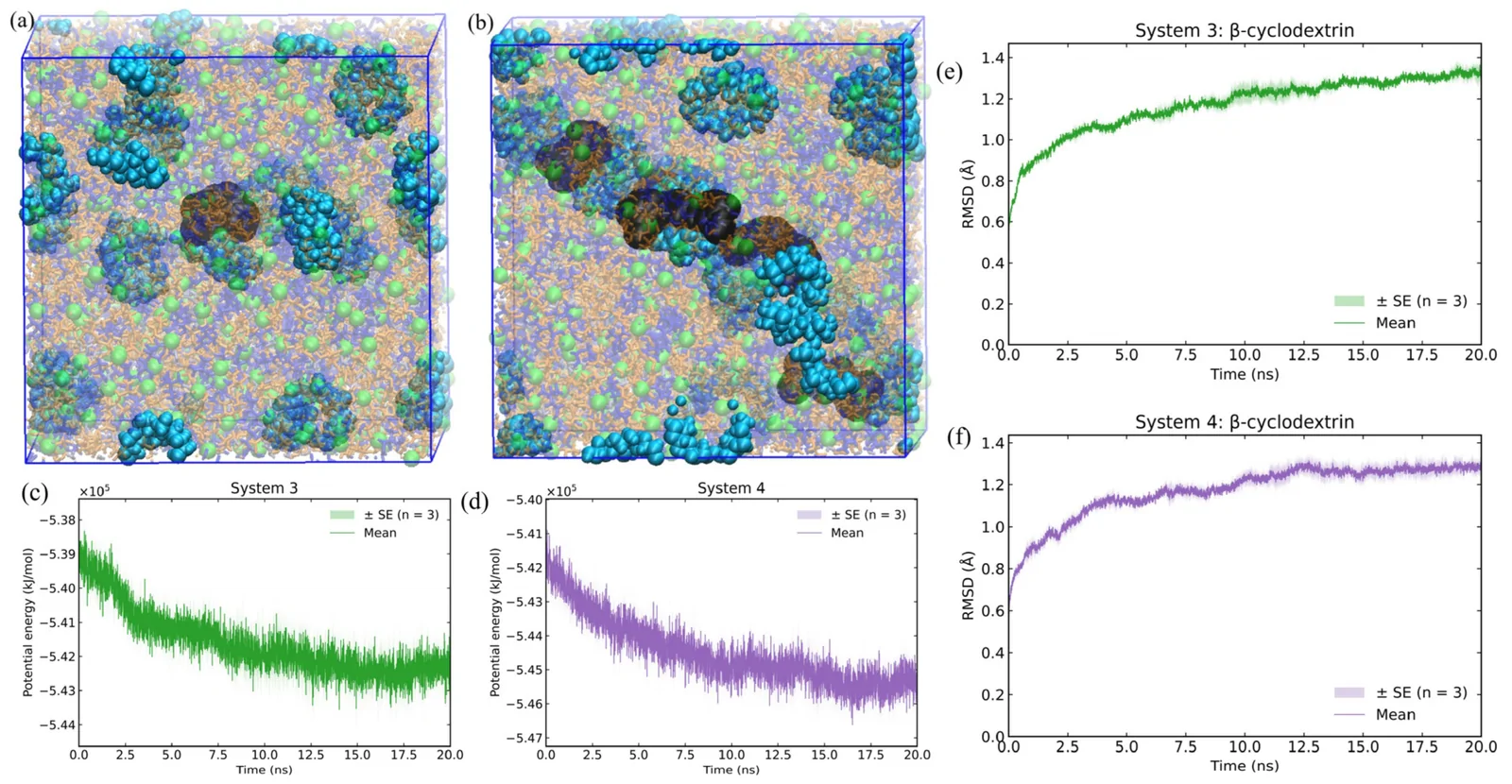
Molecular snapshots of (**a**) one molecule of curcumin in ChCl:Gly: βCD based supraDES (system 3) and (**b**) five molecules of curcumin in ChCl:Gly: βCD based supraDES (system 4). Schematic representation and color key: Licorice representation and blue (color ID: 24, transparent) molecules (ChCl); Licorice representation and orange (color ID: 3, transparent) molecules (Gly); Van der Waals surface representation and green (color ID: 7, transparent) atoms (Chloride ion); Licorice representation and ice blue (color ID: 15, transparent) molecules (Water), Van der Waals surface representation and cyan (color ID: 22) molecules (βCD), and Van der Waals surface representation and black (color ID: 15) molecules (curcumin). (**c**) Potential energy of system 3, (**d**) Potential energy of system 4, (**e**) RMSD of βCD for system 3, and (**f**) RMSD of βCD for system 4 over 20 ns of classical all-atom MD production run.

Figure 3a,b shows representative snapshots of the supraDES containing one (system 3) and five (system 4) curcumin molecules, corresponding to curcumin concentrations of approximately 4.4 and 22 mM (0.13 and 0.64 wt%) and curcumin:βCD molar ratios of 1:15 and 1:3, respectively. In both systems, the curcumin molecules are located next to βCD molecules and are surrounded by choline, glycerol, chloride and water. In system 4, the five curcumin molecules are dispersed in the box, each adjacent to a βCD molecule, and no stacked curcumin clusters are visible. As with Figure 1, these snapshots are qualitative; the curcumin–βCD proximity and the absence of curcumin aggregation are quantified in Section 2.2.4 and Section 2.2.6.

#### 2.2.2. Potential Energies

The potential energies of systems 3 and 4 (Figure 3c,d) follow the same behaviour as those of systems 1 and 2: a decrease during approximately the first 10 ns of the production run, followed by a plateau. Over the last 5 ns, the average potential energies were −542,411 ± 259 kJ/mol (system 3) and −545,407 ± 108 kJ/mol (system 4), with residual drifts of +44 and +5 kJ/mol per ns (≤0.01% per ns). The last 5 ns were therefore used for all analyses of the curcumin-containing systems.

#### 2.2.3. RMSD

The RMSD of βCD in systems 3 and 4 (Figure 3e,f) increases to approximately 1.1 Å within the first 5 ns and then levels off, reaching 1.30 ± 0.01 Å (system 3) and 1.27 ± 0.03 Å (system 4) over the last 5 ns, with residual slopes of 0.011 and 0.005 Å/ns. These values are lower and flatter than in system 2 (1.72 ± 0.07 Å; 0.030 Å/ns). However, only one (system 3) or five (system 4) of the 15 βCD molecules are in contact with curcumin, so this difference cannot be attributed to direct curcumin–βCD contacts alone, and it is reported here as an observation. In all supraDES systems, the RMSD remains below 2 Å, indicating that βCD retains its ring conformation.

#### 2.2.4. RDFs

**Figure 4:**
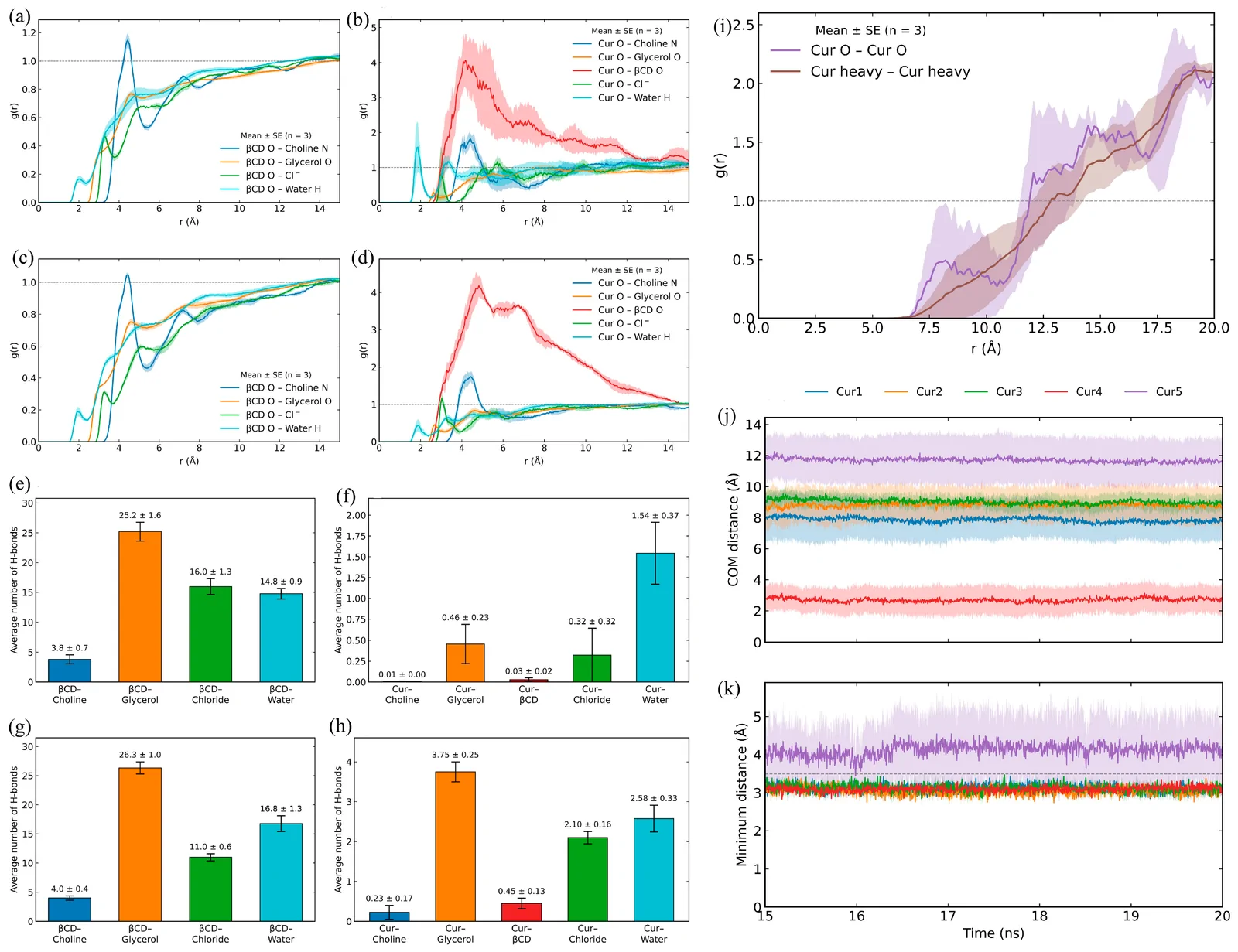
(**a**) RDF between βCD and Choline/Gly/chloride ion/water molecules in system 3. (**b**) RDF between curcumin and Choline/Gly/βCD/chloride ion/water molecules in system 3. (**c**) RDF between βCD and Choline/Gly/chloride ion/water molecules in system 4. (**d**) RDF between curcumin and Choline/Gly/βCD/chloride ion/water molecules in system 4. (**e**) Number of hydrogen bonds between βCD and Choline/Gly/chloride ion/water molecules in system 3. (**f**) Number of hydrogen bonds between curcumin and Choline/Gly/βCD/chloride ion/water molecules in system 3. (**g**) Number of hydrogen bonds between βCD and Choline/Gly/chloride ion/water molecules in system 4. (**h**) Number of hydrogen bonds between curcumin and Choline/Gly/βCD/chloride ion/water molecules in system 4. (**i**) RDF between curcumin molecules in system 4. (**j**) COM distance between curcumin and βCD in system 4. (**k**) Minimum distance between curcumin and βCD in system 4. Analysis was performed over last 5 ns of classical all-atom MD production run.

The RDFs between βCD and the supraDES components in systems 3 and 4 (Figure 4a,c) are nearly identical to those in system 2: the βCD O–water H peak at approximately 2.0 Å, the glycerol O shoulder at approximately 2.8 Å, the chloride peak at approximately 3.3 Å and the choline N peak at 4.43 Å (g(r) = 1.15 ± 0.05 in system 3 and 1.05 ± 0.02 in system 4). The presence of one or five curcumin molecules therefore does not measurably change the solvation structure of βCD, which is expected because curcumin is in contact with only a small fraction of the βCD surface in the box.

The RDFs of curcumin with the supraDES components (Figure 4b,d) were computed using the curcumin oxygen atoms (phenolic, methoxy and carbonyl) as reference. In system 3, the shortest-range feature is the curcumin O–water H peak at 1.88 Å (g(r) ≈ 1.6 ± 0.7), corresponding to water hydrogen bonded to curcumin. Chloride shows a small peak near 3.0 Å, and choline N a peak at 4.43 Å (g(r) = 1.81 ± 0.16). The most intense correlation is with βCD O, with a broad maximum at 4.18 Å (g(r) = 4.07 ± 0.77). In system 4, the same features are present, with better statistics owing to the five curcumin molecules: a glycerol O peak at approximately 2.7 Å, a chloride peak at 3.03 Å (g(r) = 1.17 ± 0.12), a choline N peak at 4.43 Å (g(r) = 1.75 ± 0.13), and a broad curcumin O–βCD O band with a maximum at 4.83 Å (g(r) = 4.18 ± 0.27) extending to approximately 7 Å. The larger uncertainties in system 3 reflect the statistics of a single curcumin molecule.

The high curcumin–βCD g(r) values show that curcumin is found near βCD more often than expected for a random distribution, i.e., the two are spatially correlated. Two points should, however, be considered when interpreting this correlation. First, the curcumin O–βCD O correlation has no peak at hydrogen-bonding distances (approximately 2.7–3.0 Å); its maximum lies at 4.2–4.8 Å, indicating that the curcumin–βCD contacts are not mediated mainly by hydrogen bonds. Second, g(r) is normalized by the bulk number density of the target atoms, which is low for βCD (15 molecules in the box); a moderate number of curcumin–βCD contacts therefore produces a high g(r). The RDF alone cannot distinguish between different association geometries (e.g., contact with the βCD rims or exterior surface, or proximity in a crowded medium). The curcumin–βCD association is therefore further characterized by distance analysis (Section 2.2.6) and interaction energies (Section 2.2.7).

To assess curcumin self-association, curcumin–curcumin RDFs were computed for system 4 using the curcumin oxygen atoms and all curcumin heavy atoms (Figure 4i). Both RDFs are zero below approximately 6.5 Å in all three replicas, and the coordination number of curcumin oxygens within 10 Å is only 0.07, showing that no direct curcumin–curcumin contacts occurred during the analysis window. The five curcumin molecules therefore remain individually dispersed in the supraDES rather than self-aggregating. The large uncertainties and the deviation of g(r) from unity at long range result from the small number of curcumin molecules (five per box) and are not interpreted further.

#### 2.2.5. Number of Hydrogen Bonds

The hydrogen-bond numbers of βCD in systems 3 and 4 (Figure 4e,g) show the same ranking as in system 2: glycerol (25.2 ± 1.6 and 26.3 ± 1.0) > water (14.8 ± 0.9 and 16.8 ± 1.3) and chloride (16.0 ± 1.3 and 11.0 ± 0.6) > choline (3.8 ± 0.7 and 4.0 ± 0.4). The βCD–chloride count varies between 10.6 and 16.0 across systems 2–4 without a systematic trend with curcumin content and is therefore attributed to sampling variability rather than to an effect of curcumin.

The hydrogen bonds of curcumin (Figure 4f,h) are formed mainly with the solvent components. In system 3, the single curcumin molecule forms 1.54 ± 0.37 hydrogen bonds with water, 0.46 ± 0.23 with glycerol, 0.32 ± 0.32 with chloride, and essentially none with βCD (0.03 ± 0.02) or choline (0.01). The large uncertainties reflect differences between replicas; for example, curcumin–chloride hydrogen bonds were observed in only one replica. In system 4, the five curcumin molecules together form 3.75 ± 0.25 hydrogen bonds with glycerol, 2.58 ± 0.33 with water, 2.10 ± 0.16 with chloride, 0.45 ± 0.13 with βCD and 0.23 ± 0.17 with choline, i.e., 0.75, 0.52, 0.42, 0.09 and 0.05 per curcumin molecule. Hence, curcumin forms fewer than 0.1 hydrogen bonds per molecule with βCD, and its hydroxyl and carbonyl groups are hydrogen bonded predominantly to glycerol, water and chloride.

#### 2.2.6. Distance Between Curcumin–βCD

To characterize the curcumin–βCD association beyond the RDF, the distance between the center of mass (COM) of each curcumin molecule and the COM of the nearest βCD, as well as the minimum heavy-atom distance between each curcumin and any βCD, were monitored over the last 5 ns of each replica of system 4 (Figure 4j,k). Each curcumin molecule remained associated with the same βCD molecule throughout the analysis window in all replicas, except for one curcumin that alternated between two neighbouring βCD molecules in two replicas. The minimum distances of curcumin molecules 1–4 fluctuated around 3.1 Å (3.05–3.12 Å on average), and contact (minimum heavy-atom distance ≤ 4.5 Å) was maintained in 100% of the frames in all replicas. Curcumin 5 was in contact with βCD in two replicas but not in the third (average minimum distance 5.9 Å), giving an average of 4.1 ± 0.9 Å. Thus, 14 of the 15 curcumin–βCD pairs (five molecules × three replicas) were in persistent contact during the analysis window.

The COM distances differ markedly between the curcumin molecules: 2.7 ± 0.9 Å for curcumin 4, 7.9 ± 1.3 Å, 8.8 ± 1.2 Å and 9.0 ± 0.5 Å for curcumins 1–3, and 11.7 ± 1.6 Å for curcumin 5. Combined with minimum distances of approximately 3 Å, the larger COM separations indicate that curcumins 1–3 are in contact with the rims or exterior surface of βCD, whereas curcumin 4 is located close to the βCD centre of mass. These data establish that curcumin–βCD association in system 4 is persistent on the time scale of the analysis window. Because the analysis covers the last 5 ns, it cannot exclude that the associations originate from earlier stages of the trajectories, and it does not provide equilibrium residence times. A detailed characterization of the host–guest geometry (position relative to the cavity axis, penetration depth, orientation and binding free energy) would require dedicated analyses and enhanced-sampling methods and is beyond the scope of this work, which focuses on intermolecular interactions. Accordingly, no inclusion complex formation is claimed here.

#### 2.2.7. Interaction Energies

The interaction energies of βCD with the supraDES components in systems 3 and 4 (Table 4) are very similar to those in system 2 (Table 3). For example, the βCD–glycerol electrostatic and van der Waals energies are −992.6 ± 42.6 and −3473.7 ± 65.2 kJ/mol in system 3 and −1082.4 ± 63.0 and −3329.6 ± 58.6 kJ/mol in system 4, compared with −1010.1 ± 21.9 and −3273.3 ± 139.5 kJ/mol in system 2. The differences between systems 2–4 are small and not monotonic with curcumin content, confirming that the interaction of βCD with the DES components is not significantly altered by curcumin at the concentrations studied.

The interaction energies of curcumin with the supraDES components are summarized in Table 5. As for the other tables, the values are totals; in system 4 they are summed over all five curcumin molecules. Normalized per curcumin molecule, the electrostatic and van der Waals energies are −33.2 and −87.1 kJ/mol (system 3) and −37.8 and −79.1 kJ/mol (system 4) with glycerol; −22.0 and −65.2 and −21.7 and −74.0 kJ/mol with choline; −42.5 and −3.3 and −54.6 and −3.2 kJ/mol with chloride; −61.5 and +2.4 and −24.1 and −7.3 kJ/mol with water; and −5.5 and −36.1 and −7.7 and −49.9 kJ/mol with βCD. The total short-range interaction energy per curcumin molecule is thus approximately −354 kJ/mol in system 3 and −359 kJ/mol in system 4, of which βCD contributes only 12% and 16%, respectively. Curcumin interacts most strongly with glycerol (van der Waals and electrostatic), with chloride (electrostatic) and with choline (van der Waals). The single curcumin molecule in system 3 interacts more strongly with water than the curcumin molecules in system 4, consistent with its larger number of hydrogen bonds with water.

The curcumin–βCD interaction energies should be reconciled with the pronounced curcumin–βCD RDF correlation. First, the curcumin–βCD interaction is dominated by van der Waals contributions (van der Waals-to-electrostatic ratio of approximately 6.5 in both systems). This is consistent with the absence of a hydrogen-bonding peak in the curcumin O–βCD O RDF and with fewer than 0.1 curcumin–βCD hydrogen bonds per curcumin. Second, as noted above, the high g(r) arises partly from the low bulk density of βCD in the box and therefore does not imply a large number of strong contacts. Third, the modest per-molecule value is not caused by averaging over non-contacting molecules: in system 4, 14 of the 15 curcumin–βCD pairs remained in contact during the analysis window (Section 2.2.6). Each curcumin molecule thus contacts βCD through only a limited part of its surface, while the rest of the molecule is solvated by the much more abundant DES components and water. Finally, pairwise interaction energies are not binding free energies. The association of curcumin with βCD in solution also depends on the partial desolvation of both molecules, on entropic contributions and on competition with the solvent components, which can only be evaluated by free-energy methods such as umbrella sampling or metadynamics. Such calculations were not performed here. We therefore describe the curcumin–βCD relationship as a persistent, van der Waals-dominated spatial association, and we do not draw conclusions regarding curcumin stabilization or cooperative binding.

#### 2.2.8. Density Profiles

**Figure 5:**
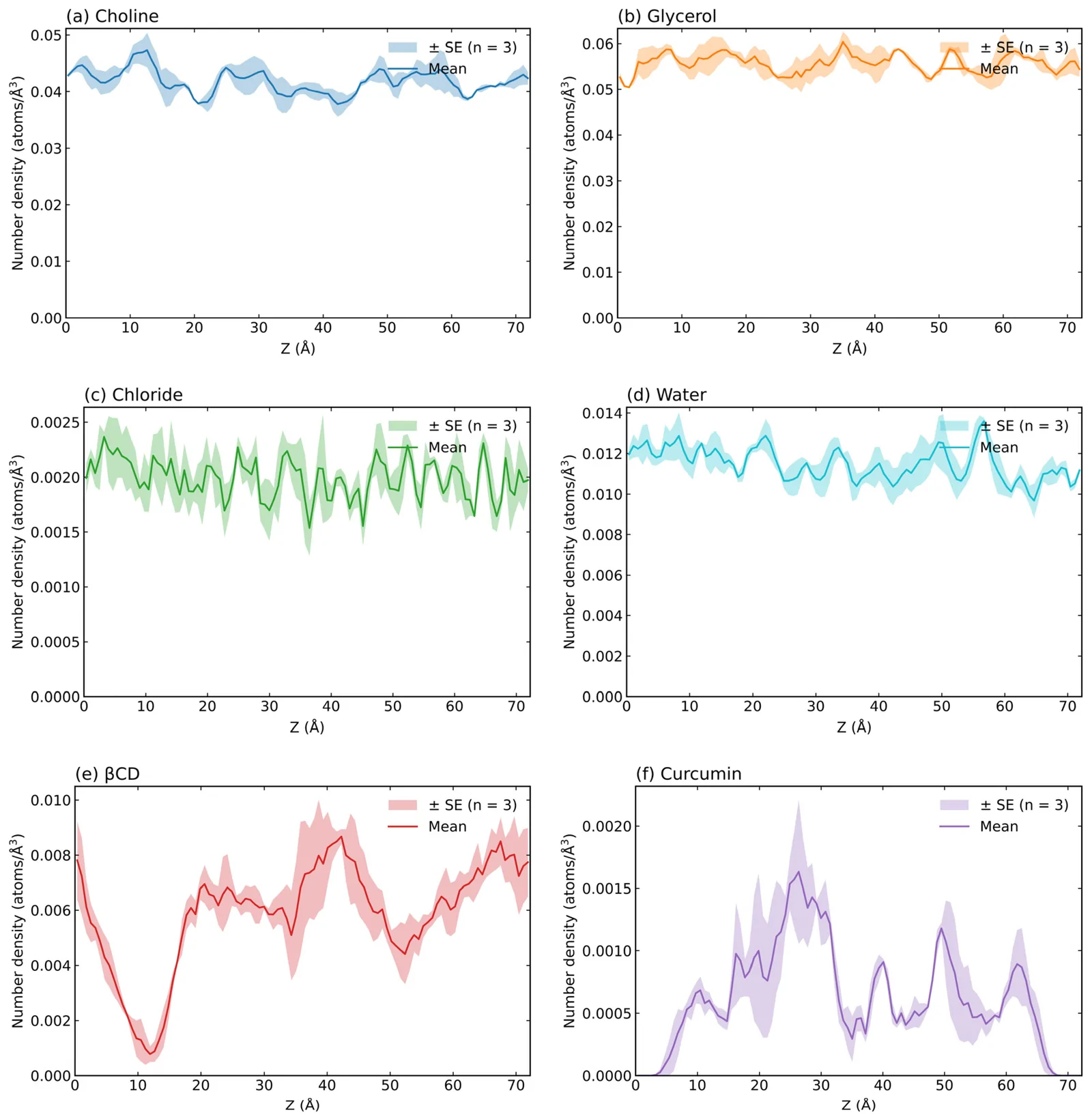
Density profiles along the *Z*-axis for (**a**) Choline, (**b**) Gly, (**c**) chloride ion, (**d**) water molecules, (**e**) βCD, and (**f**) curcumin in system containing five molecules of curcumin in ChCl:Gly:βCD based supraDES. Analysis was performed over last 5 ns of classical all-atom MD production run.

Figure 5 shows the number-density profiles along the *Z*-axis of the box for all components of system 4, averaged over the last 5 ns and three replicas (100 bins). The profiles of choline, glycerol, chloride and water are essentially flat, with coefficients of variation of 5%, 4%, 9% and 7%, respectively. This indicates that the DES components and water are homogeneously distributed, without layering or microphase separation; in particular, water does not form segregated domains. In contrast, the βCD and curcumin profiles are non-uniform (coefficients of variation of 33% and 65%), because they are determined by the positions of only 15 βCD and five curcumin molecules.

The one-dimensional profiles of curcumin and βCD are not significantly correlated (Pearson r ≈ −0.04 for the replica-averaged profiles), because a projection onto the *Z*-axis averages over the X–Y plane and cannot resolve local molecular contacts. The density profiles therefore establish only that curcumin is not segregated into any particular region of the box and that the solvent remains homogeneous around it, i.e., they reflect spatial partitioning rather than direct molecular association. Local curcumin–βCD association was assessed instead by the RDF and distance analyses (Section 2.2.4 and Section 2.2.6).

Overall, the present simulations describe curcumin in the supraDES as a solute that is solvated mainly by glycerol, chloride and water through hydrogen bonding and electrostatic interactions, and by choline and glycerol through van der Waals interactions, while being spatially associated with βCD through predominantly van der Waals contacts. These results should be compared with the experimental studies summarized in Table 1 with care. Those studies characterize host–guest complexation in dilute aqueous media [21,22,23,24,25,26,27,28,29,30,31,32,33,34,35,36], whereas the present work evaluates intermolecular interactions in a crowded, hydrogen-bonded eutectic medium and does not quantify inclusion geometry or binding free energy. Two qualitative points are nevertheless consistent with the experimental literature. First, experimental studies show that components that compete for the host or increase guest solvation weaken cyclodextrin complexation, as observed for urea [24] and for ionized, more strongly hydrated guests [31,32,33,34]. Similarly, curcumin here forms most of its hydrogen bonds and its strongest electrostatic interactions with glycerol, chloride and water rather than with βCD, indicating that the DES components compete effectively with βCD for curcumin. Second, neutral guests are generally bound more strongly by βCD than their charged forms [21,26,31,32]. The neutral diketo form of curcumin considered here is therefore the relevant form for its interactions with βCD, whereas ionized forms of curcumin may be more strongly solvated by the polar DES components. Whether, and to what extent, curcumin forms inclusion complexes with βCD in this supraDES remains to be established by dedicated host–guest analyses, enhanced-sampling free-energy calculations and experiments.

## 3. Materials and Methods

### 3.1. Theoretical Model and Designed System

**Figure 6:**
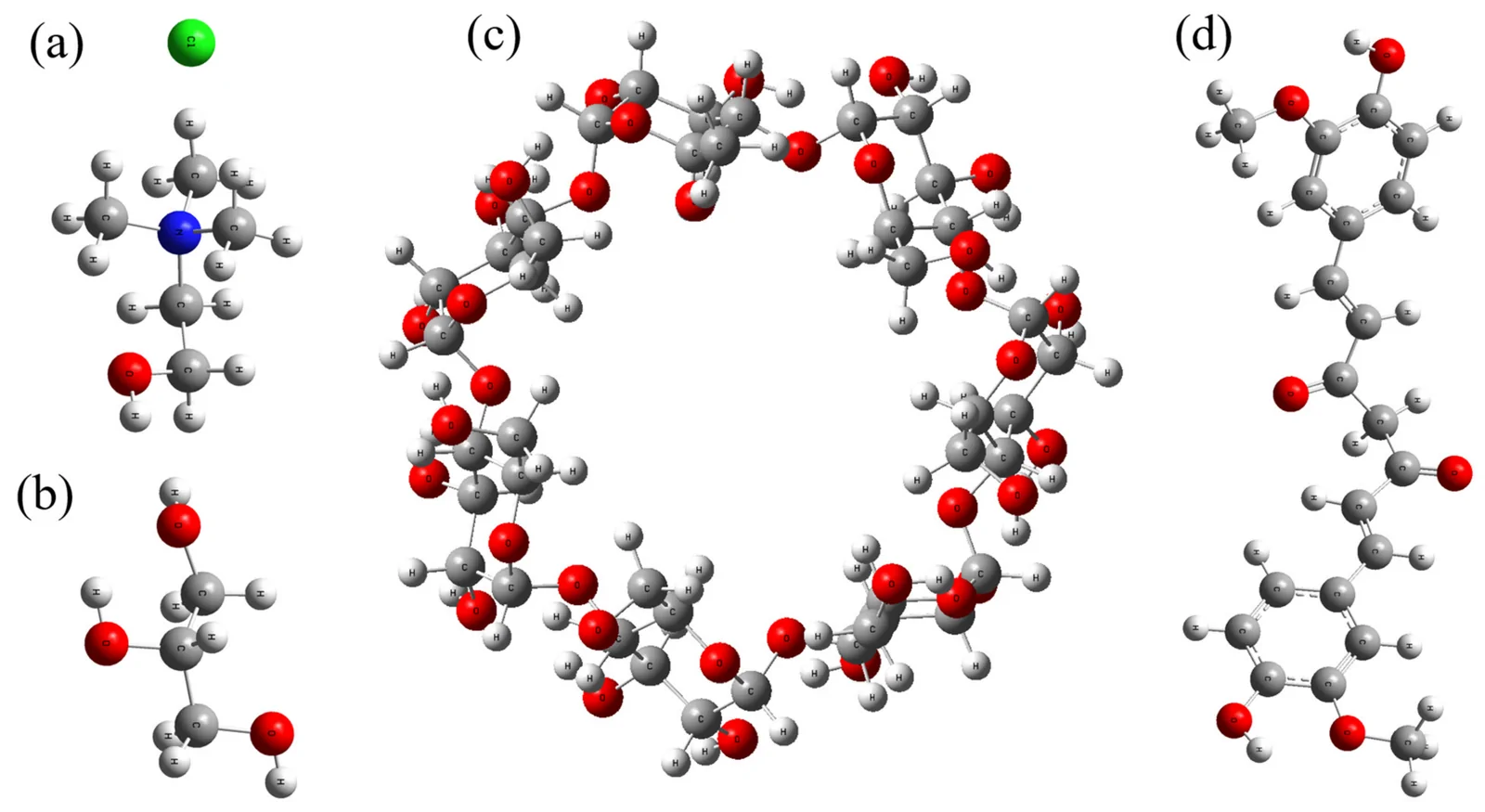
Chemical structures (3D) of (**a**) ChCl, (**b**) Gly, (**c**) βCD and (**d**) curcumin. Color key: grey (carbon); white (hydrogen); blue (nitrogen); red (oxygen); green (chloride).

In this study, curcumin was selected as theoretical curcuminoid model, while ChCl, Gly, and βCD were chosen as the supraDES components. Water molecules were added as the solvent to represent the theoretical model of 10% water content (*w*/*w*) in supraDES components for classical all-atom MD simulations (Figure 6, Table 6). These models were inspired by previously reported experimental studies [15,37,38]. This work is done via computational perspectives, enabling a molecular-level investigation of the formation mechanism of supraDES, and intermolecular interactions between curcumin and supraDES.

Table 6 lists the composition of the four classical all-atom MD simulated systems. System 1 represents the pure ChCl:Gly (1:2) DES, while System 2 incorporates βCD and water to form the supramolecular DES. Systems 3 and 4 contain one and five curcumin molecules, respectively, to examine the interactions of curcumin with the supramolecular DES. The one and five curcumin molecules correspond to curcumin concentrations of approximately 4.4 and 22 mM (0.13 and 0.64 wt%), i.e., curcumin:βCD molar ratios of 1:15 and 1:3, respectively.

Curcumin exists in keto–enol tautomeric equilibrium, and the two forms differ in planarity and hydrogen-bonding capability. In this study, curcumin was modelled in its diketo form, in which the central methylene group separates two free carbonyl groups. Both phenolic hydroxyl groups were kept protonated, giving a neutral molecule (C_21_H_20_O_6_, net charge 0). This choice reflects reports that the diketo tautomer predominates in neutral and acidic aqueous media [22,23,24,25,26,27,28,29,30], which corresponds to the water-containing supraDES system studied in this work. βCD was likewise modelled as neutral, with all primary and secondary hydroxyl groups protonated, since its hydroxyls deprotonate only under strongly alkaline conditions (pKa ≈ 12) [28,29,30,31,32,33,34,35,36]. We note that the keto-enol tautomer, which is favoured in many organic solvents and in the solid state, forms an intramolecular O–H···O=C hydrogen bond and adopts a more planar geometry. It may therefore interact differently with the supraDES components, which should be considered when interpreting the present results.

Table 7 summarizes the equilibrated box dimensions and computed densities of the four simulated systems. The computed density of the neat ChCl:Gly (1:2) system agrees well with the experimental value, confirming that the adopted force field reproduces the bulk packing of the DES. The densities of the βCD- and curcumin-containing systems are consistent with values reported for related aqueous ChCl:Gly systems, for which no experimental data at the exact compositions are available [39,40,41].

### 3.2. Classical All-Atom MD Simulation

For the classical all-atom MD simulations, ChCl, Gly, and βCD in a 1:2:0.02 ratio with 10% water content (*w*/*w*) were employed as the supraDES, corresponding to 750 ChCl, 1500 Gly, 15 βCD, and 1443 water molecules. Additionally, 1 and 5 molecules of curcumin were added to study its interaction with the supraDES.

Classical all-atom MD simulations were performed to investigate the intermolecular interactions and dynamic behavior of curcumin in the presence of the supraDES. The initial topology and force field parameters for ChCl, Gly, βCD, and curcumin were obtained from the Automated Topology Builder database [42], ensuring compatibility with the GROMOS54A7 force field [43]. Water molecules were modeled using the SPC/E water model [44], in which partial charges are assigned to oxygen and hydrogen atoms to reproduce realistic hydrogen-bonding and dielectric properties. A cubic simulation box measuring 8.5 × 8.5 × 8.5 nm^3^ was constructed, containing the supraDES and curcumin molecules, as described above. The initial configurations underwent energy minimization until the maximum force was reduced below 1000 kJ mol^−1^ nm^−1^, thereby enhancing system stability. Equilibration was carried out in two stages: first, an NVT ensemble simulation at 298 K for 0.1 ns to stabilize the temperature, followed by an NPT ensemble simulation at 298 K and 1 bar for 0.1 ns to stabilize the pressure. Subsequently, a 20 ns production run was performed, during which the output analysis was evaluated.

To maintain molecular geometry, bond lengths were constrained using the LINCS algorithm. A cutoff distance of 1.0 nm was applied for short-range interactions, while long-range electrostatic interactions were calculated using the Particle Mesh Ewald [45] method with a grid spacing of 0.16 nm. The Nosé-Hoover thermostat [46] was employed to maintain the temperature at 298 K, while the Berendsen barostat [47] was used to control the pressure at 1 bar. Periodic boundary conditions were applied in all spatial dimensions to mimic an infinite system. All simulations were performed using GROMACS (version 2021.4) [48]. Molecular snapshots, RDFs, number of hydrogen bonds, interaction energies, potential energy, density profiles, and RMSD were analyzed using VMD (version 1.9.1) software [49] and Python (version 3.12), respectively. Three independent classical all-atom MD simulations were performed for each system, starting from different initial configurations, and the reported properties are given as mean ± standard error (SE = SD/√3, *n* = 3).

All analyses were performed over the last 5 ns (15–20 ns) of each production run, after the potential energy had levelled off (Figure 1 and Figure 3). RDFs were computed up to 15 Å with a bin width of 0.05 Å (up to 20 Å with a bin width of 0.13 Å for the curcumin–curcumin RDFs), and coordination numbers were obtained by integrating the RDFs up to their first minimum. Hydrogen bonds were identified using a donor–acceptor distance cut-off of 3.5 Å and a donor–hydrogen···acceptor angle cut-off of 150°. Short-range electrostatic and van der Waals interaction energies were obtained from the energy-group decomposition in GROMACS, with energy groups defined as whole molecule types (choline, chloride, glycerol, water, βCD and curcumin), and are reported as total energies between the respective groups. For system 4, the distance between the centre of mass of each curcumin molecule and that of the nearest βCD, and the minimum heavy-atom distance between each curcumin and any βCD, were calculated for every frame. Density profiles were calculated along the *Z*-axis using 100 bins, and the RMSD of βCD was calculated relative to its starting structure.

## 4. Conclusions

Classical all-atom MD simulations were employed to elucidate the molecular mechanisms governing the formation of the ChCl:Gly:βCD supraDES and its subsequent interactions with curcumin. Molecular snapshots, RDFs, hydrogen bond analysis, interaction energies, density profiles, and RMSD were used to analyze the simulation trajectories and characterize the structural and intermolecular interactions within the systems.

Regarding supraDES formation, the potential energies of all systems levelled off after approximately 10 ns, and the three independent replicas reached the same energetic state, so the last 5 ns of each trajectory were used for analysis. The incorporation of βCD and water preserved the main structural motifs of the ChCl:Gly DES, namely O–H···Cl^−^ hydrogen bonding between glycerol and chloride (RDF peak at 2.9–3.0 Å) and choline–chloride ion pairing (4.4–4.5 Å). However, it reduced the first-shell coordination numbers by 10–12% and the numbers of choline–glycerol, glycerol–chloride and choline–chloride hydrogen bonds by 32%, 11% and 9%, respectively. It also weakened the DES–DES short-range interaction energies by 15–31%, as water and βCD partly replace DES–DES contacts. These changes reflect the combined effect of βCD and water. βCD retained its ring conformation (RMSD < 2 Å) and was solvated mainly by glycerol (approximately 1.7 hydrogen bonds per βCD), followed by water and chloride, whereas choline interacted with βCD mainly through van der Waals contacts.

Regarding the interactions of the supraDES with curcumin, curcumin was spatially correlated with βCD (curcumin O–βCD O RDF maximum at 4.2–4.8 Å). In the system with five curcumin molecules, 14 of the 15 curcumin–βCD pairs (five molecules × three replicas) remained in contact during the analysis window, and no curcumin–curcumin contacts occurred (curcumin–curcumin g(r) = 0 below approximately 6.5 Å), i.e., curcumin did not self-aggregate. However, the curcumin–βCD association is dominated by van der Waals interactions, involves fewer than 0.1 hydrogen bonds per curcumin, and accounts for only 12–16% of the total short-range interaction energy of curcumin. Most of this energy, and most of the hydrogen bonds of curcumin, involve glycerol, chloride, choline and water. The presence of curcumin did not measurably change the solvation of βCD by the DES components. These conclusions concern intermolecular interactions only: pairwise interaction energies are not binding free energies, and the host–guest inclusion geometry, the binding thermodynamics and the chemical stability of curcumin were not addressed. These aspects require experimental studies and enhanced-sampling free-energy calculations.

## Figures and Tables

**Table 1:** Previous experimental studies on the effects of cyclodextrin structure and ionization state on host–guest complex formation and aqueous solubility.

Host(s)	Guest	Conditions/Ionization State	Main Methods	Key Findings	Ref.
α-, β-, γ-CD	Baricitinib	pH 1.6 (cation) vs. 6.8 (neutral); 290–310 K	Phase solubility, ^1^H NMR/ROESY, CE	1:1 complexes; β-CD best solubilizer; neutral form bound ~2–3× more strongly than cation; enthalpy-driven	[21]
α-, β-, γ-CD, DM-β-CD	Amino acids	Protonated guests	ESI-MS, CID, molecular mechanics	β-, γ-CD > α-CD; DM-β-CD markedly improved binding; aromatic > aliphatic	[22]
α-, β-, γ-CD	C_5_–C_9_ n-alkanes	Neutral; water, 25 °C	Volatilization rate	β-, γ-CD 1:1; α-CD 1:1 and 2:1; K increases with chain length	[23]
β-, γ-CD	Coumarin dye (7-DCCAE)	Water, ±7 M urea	UV–vis, fluorescence, ^1^H NMR, ITC	1:1; K increases with cavity size (ITC: 4410 → 8350 M^−1^); urea weakens binding	[24]
α-, β-, HP-β-, M-β-CD	Valsartan	±Tween 80	Phase solubility, NMR/ROESY, calorimetry, docking/MD	M-β-CD most effective; 1:1; Tween 80 raises K_1_ (1742 → 1992 M^−1^)	[25]
21 native and modified CDs	Aspartame	pH 2.5, 5.2, 9.0 (cation, zwitterion, anion)	^1^H NMR, CE, ROESY, modeling	β-CD family best; acetyl-β-CD (DS 7) most stable; stability depends on charge state	[26]
β-, γ-, HP-β-, Me-β-CD	Piroxicam	Several pH values and temperatures	Phase solubility, van ’t Hoff	A_L_ (1:1) for β-, γ-, HP-β-CD; A_P_ for Me-β-CD; K: β-CD > γ-CD > HP-β-CD; strong pH effect	[27]
β-, HP-β-, SBE-β-CD	Dofetilide	pH 2.0 vs. 7.4	Phase solubility, solid dispersions, FTIR, PXRD, DSC	At pH 7.4 solubility ×3.6, ×11.8, ×18.1; little gain at pH 2.0 (ionized drug)	[28]
β-, RM-β-, HP-β-, SBE-β-CD	Four tyrosine kinase inhibitors	Mainly pH 2.0	Phase solubility, Job plot, ROESY, docking	1:1; β-CD cavity fits all guests; SBE-β-CD up to ~200-fold (lapatinib)	[29]
β-, HP-β-CD vs. PAMAM dendrimers	Niclosamide	pH 4, 7, 11 and water	Phase solubility, dissolution, DSC, XRPD	Dendrimers bound more strongly than CDs; CDs gave faster release	[30]
β-CD	Ibuprofen (+10 literature datasets)	pH 2.5–5.5; 15–55 °C	ITC, UV–vis pKa titration	K decreases with ionization; predictive solubility constants across pH expression was proposed	[31]
β-, HP-β-CD	Ketoprofen	pH 3.0, 6.0, 7.4	Phase solubility, FTIR, XRD, DFT	K falls 639 → 9.8 M^−1^ (β-CD) and 1394 → 29.6 M^−1^ (HP-β-CD); enthalpy-driven	[32]
β-CD	Aspirin	Neutral vs. anionic	Ultrasonic relaxation	Charge raises dissociation rate; association diffusion-controlled	[33]
α-CD	Aminobenzoic acids	Different pH; Na^+^/K^+^ salts	^1^H NMR, calorimetry	Salt effects depend on guest ionization (anion vs. zwitterion) and hydration	[34]
TRIMEB	Midazolam	Open-ring (cationic) vs. closed-ring (neutral)	Docking, QM, cell viability	Open-ring form binds better (ΔG_bind_ = −5.57 kcal/mol)	[35]
Amino-, thiol-, phenol-functionalized β-CDs	PFAS	pH 7 vs. 12 (host ionization)	^19^F NMR titration	K reduced by 12–98% at alkaline pH	[36]

Abbreviations: CD, cyclodextrin; DM-β-CD, heptakis(2,6-di-O-methyl)-β-CD; M-β-CD/Me-β-CD, methyl-β-CD; HP-β-CD, 2-hydroxypropyl-β-CD; SBE-β-CD, sulfobutylether-β-CD; RM-β-CD, randomly methylated β-CD; TRIMEB, heptakis(2,3,6-tri-O-methyl)-β-CD; PAMAM, polyamidoamine; PFAS, per- and polyfluoroalkyl substances; CE, capillary electrophoresis; ESI-MS, electrospray ionization mass spectrometry; CID, collision-induced dissociation; ITC, isothermal titration calorimetry; ROESY, rotating-frame Overhauser effect spectroscopy; K, binding (stability) constant.

**Table 2:** Average short-range electrostatic and van der Waals interaction energies between choline and chloride, choline and glycerol, and chloride and glycerol in the DES and supraDES systems. Values are total short-range energies between the two molecular groups (not normalized per molecule), given as mean ± SE of three independent replicas over the last 5 ns.

Interaction Energy (kJ/mol)	DES (System 1)		SupraDES (System 2)	
	Electrostatic	Van Der Waals	Electrostatic	Van Der Waals
Choline and glycerol	−33,898 ± 292	−33,746 ± 126	−23,495 ± 179	−28,348 ± 48
Choline and chloride	−85,448 ± 290	1699 ± 90	−69,781 ± 739	727 ± 36
Chloride and glycerol	−153,273 ± 359	20,817 ± 54	−129,730 ± 1182	17,225 ± 190

**Table 3:** Average short-range electrostatic and van der Waals interaction energies of βCD with the major components of the supraDES systems. Values are total short-range energies between the two molecular groups (not normalized per molecule), given as mean ± SE of three independent replicas over the last 5 ns.

Interaction Energy (kJ/mol)	SupraDES (System 2)	
	Electrostatic	Van Der Waals
Between βCD and choline	−410.2 ± 10.2	−2073.9 ± 43.4
Between βCD and glycerol	−1010.1 ± 21.9	−3273.3 ± 139.5
Between βCD and chloride	−1166.4 ± 62.2	−235.1 ± 0.5
Between βCD and water	−532.9 ± 27.0	−374.6 ± 11.0

**Table 4:** Average short-range electrostatic and van der Waals interaction energies of βCD with the major components of the simulated system. Values are total short-range energies between the two molecular groups (not normalized per molecule), given as mean ± SE of three independent replicas over the last 5 ns.

Interaction Energy (kJ/mol)	SupraDES + One Curcumin (System 3)		SupraDES + Five Curcumin (System 4)	
	Electrostatic	Van Der Waals	Electrostatic	Van Der Waals
Between βCD and choline	−421.5 ± 44.9	−2323.3 ± 46.3	−449.9 ± 19.4	−2122.2 ± 59.4
Between βCD and glycerol	−992.6 ± 42.6	−3473.7 ± 65.2	−1082.4 ± 63.0	−3329.6 ± 58.6
Between βCD and chloride	−1504.0 ± 48.7	−264.0 ± 3.5	−1202.4 ± 38.0	−236.8 ± 10.8
Between βCD and water	−591.4 ± 59.0	−457.7 ± 31.3	−660.9 ± 45.3	−430.2 ± 4.8

**Table 5:** Average short-range electrostatic and van der Waals interaction energies of Curcumin with the major components of the simulated system. Values are total short-range energies between the two molecular groups (not normalized per molecule), given as mean ± SE of three independent replicas over the last 5 ns.

Interaction Energy (kJ/mol)	SupraDES + One Curcumin (System 3)		SupraDES + Five Curcumin (System 4)	
	Electrostatic	Van Der Waals	Electrostatic	Van Der Waals
Between Curcumin and βCD	−5.5 ± 2.0	−36.1 ± 9.9	−38.7 ± 2.0	−249.3 ± 10.1
Between Curcumin and choline	−22.0 ± 6.5	−65.2 ± 13.5	−108.7 ± 8.7	−370.0 ± 31.3
Between Curcumin and glycerol	−33.2 ± 14.2	−87.1 ± 7.5	−189.0 ± 17.1	−395.3 ± 35.2
Between Curcumin and chloride	−42.5 ± 19.3	−3.3 ± 4.4	−273.2 ± 16.8	−15.9 ± 4.4
Between Curcumin and water	−61.5 ± 10.3	2.4 ± 1.8	−120.5 ± 20.0	−36.6 ± 3.1

**Table 6:** Summarizes designed systems for classical all-atom MD simulations.

	ChCl	Gly	βCD	Water	Curcumin
1	750	1500	-	-	-
2	750	1500	15	1443	-
3	750	1500	15	1443	1
4	750	1500	15	1443	5

**Table 7:** Box dimensions and computed densities of the simulated systems after equilibration, compared with experimental densities at 298 K.

	Number of Atoms	Box Size (nm^3^)	Computed Density (kg/m^3^)	Experimental Density (kg/m^3^)
1	37,500	6.93 × 6.93 × 6.93	1114.5 ± 9.2	1160–1220 [39]
2	44,034	7.28 × 7.28 × 7.28	1125.6 ± 21	1110–1290 [40]
3	44,081	7.22 × 7.22 × 7.22	1224.2 ± 1.7	1150–1300 [41]
4	44,269	7.22 × 7.22 × 7.22	1226.6 ± 5.5	1150–1300 [41]

## Data Availability

All data supporting the findings of this computational study are reported in the manuscript.

## Abbreviations

The following abbreviations are used in this manuscript:

βCD	β-cyclodextrin
ChCl	Choline chloride
ChN	Choline nitrogen
CN	Coordination numbers
COM	Center of mass
DES	Deep Eutectic Solvent
Gly	Glycerol
GlyO	Glycerol hydroxyl oxygens
MD	Molecular dynamics
RDFs	Radial distribution functions
RMSD	Root-mean-square deviation
SupraDES	Supramolecular Deep Eutectic Solvents
